# Characterizing Family Planning Utilization in Adult Women and Adolescents in Pohnpei, Federerated States of Micronesia

**DOI:** 10.1007/s10995-024-03906-6

**Published:** 2024-03-08

**Authors:** Katherine McDonald, Haley L. Cash McGinley, Delpihn Abraham, Stephanie F. Kapiriel, Marcy Lorrin

**Affiliations:** 1 College of Public Health, University of Iowa, Iowa City, USA; 2 Pacific Island Health Officers Association, Honolulu, USA; 3 Pohnpei State Department of Health and Social Services, Pohnpei, Federated States of Micronesia; 4 FSM National Department of Health and Social Affairs, Pohnpei, Federated States of Micronesia

**Keywords:** Family planning, Contraceptives, Micronesia, Pohnpei

## Abstract

**Introduction:**

Increasing family planning xutilization in low-income countries to improve health outcomes of women and children is a global priority. The Federated States of Micronesia (FSM) has poor maternal child health outcomes; therefore, this study aimed to examine family planning utilization in Pohnpei State, FSM.

**Methods:**

This cross-sectional study sought to characterize family planning utilization in adult women of reproductive age and high school age adolescents in Pohnpei using representative survey data collected in 2019 (N = 570 and N = 1726, respectively). Chi-square tests were used to determine significant factors associated with family planning utilization.

**Results:**

Among adult women of reproductive age (18–49 years old) not trying to get pregnant, 31.6% reported using contraception during last intercourse. Contraceptive use was significantly lower among younger women (18–24 years old) (21.7%, p = 0.021), unmarried women (18.6%, p < 0.001), those without health insurance (28.7%, p = 0.030), those who have never had a pap smear (20.5%, p < 0.001), and those who have never been pregnant (14.5%, p < 0.002). Among adolescents who reported being sexually active, 28.5% reported using any contraception at last intercourse and 22.6% reported using a condom at last intercourse. Condom use among sexually active adolescents was lowest among 12th graders (13.5%, p < 0.001) and girls (16.8%, p = 0.004).

**Conclusions:**

Our findings suggest that young, unmarried, never pregnant women face an unmet need for family planning. Additionally, women with lower access to and use of healthcare services have lower use of family planning.

## Significance

Data published on reproductive health in FSM have been limited. This study points to several possible interventions to increase family planning utilization in Pohnpei, including the development of a comprehensive school-based health education curriculum, increasing access to and promotion of well-woman visits, and increasing use of long-acting reversible contraception.

## Introduction

Family planning allows people to attain the number of children they desire and to determine when and how far apart their pregnancies occur. Ensuring access to family planning resources, such as contraceptives to prevent pregnancy, is essential to improving maternal and child health around the world, as outlined in the United Nation’s Sustainable Development Goals (United Nations [UN], 2020). Target 3.7 of the Sustainable Development Goals calls for universal access to family planning by 2030 and the monitoring of family planning indicators. Over the past 20 years, increasing family planning utilization has led to a 40% decrease in the number of maternal deaths in low-income countries and has improved health outcomes for children worldwide (Ahmed et al., 2012; Cleland et al., 2012). Family planning also has economic implications: it boosts savings in maternal and child healthcare and leads to better educational and economic outcomes for women (Darroch et al., 2016). However, one in ten women globally have an unmet need for family planning (Family Planning International [FPI], 2009).

The Federated States of Micronesia (FSM) is an independent Pacific Island nation in the Western Pacific Ocean located north of Papua New Guinea (Cash et al., 2021). It is made up of four states comprising of 607 small islands. According to a 2010 census, the total population of FSM was 102,843 (Federated States of Micronesia [FSM] Statistics Division, n.d.). The capital state of FSM, Pohnpei, was estimated to have a population of 36,832 in 2020 (FSM Statistics Division, n.d.). Although FSM is an independent nation, it has a compact of free association with the United States which entitles it to certain US federal programs and funding such as the Human Resources Service Administration (HRSA) Title V Maternal and Child Health Block Grant.

Similar to other Pacific Island nations, FSM has a high rate of maternal mortality, and it falls into the high-risk category for reproductive risk (FPI, 2009; United Nations International Children’s Emergency Fund [UNICEF], 2013). The rate of maternal mortality in FSM in 2017 was 88 deaths per 100,000 live births, while in the United States, the maternal mortality rate was 17.3 deaths per 100,000 live births (UNICEF, n.d.; Centers for Disease Control and Prevention [CDC], n.d.). FSM recently fell short of meeting Millennium Development Goal 5, which is to improve maternal and child health, and reproductive health is a key facet of this goal (Pacific Island Regional MDGs Tracking Report, 2011). In addition, the adolescent fertility rate was 37 per 1,000 women ages 15–19 (The World Bank, n.d.), compared to 16.7 per 1000 in the US (CDC, 2019).

Having access to family planning is important for both adults and adolescents. One previous study of youth not attending school in FSM found that 76.1% were sexually active and 88.3% rarely or never used condoms (Corner et al., 2005). Short-term health outcomes of unmet need for family planning for adolescents include unintended pregnancies and sexually transmitted infections (WHO, 2020). When compared to women 20–24 years old, adolescent girls who are pregnant have higher risk of adverse outcomes such as eclampsia, puerperal endometritis, systemic infections, low birthweight, preterm delivery, and severe neonatal conditions (Ganchimeg et al., 2014). Additionally, abortion is not available in FSM, so there is potential for pregnant girls to turn to unsafe practices if they cannot afford to travel off-island for an abortion.

The long-term impacts of unmet need for family planning include health, educational, and economic effects. In low-income countries, only 11% of adolescents who become pregnant continue with their education, and the children of adolescent parents tend to perform worse in school (Molina et al., 2010). In small island states, improved access to family planning could lead to saving in healthcare spending. One study found that in Vanuatu and the Solomon Islands, meeting family planning needs would save Vanuatu $5.19 million and the Solomon Islands $3.36 million over a fifteen-year period (Kennedy et al., 2013). Providing family planning also has other beneficial effects, such as increasing the female labor force (Bloom et al., 2009).

The data that have been published on reproductive health in FSM have been limited and mostly focused on sexual risk behavior, pregnancy, healthcare seeking, and HIV/AIDS (Smith, 2013). To understand the full picture of reproductive health, it is also important to consider the prevalence of family planning utilization, associated risk factors, and barriers to utilization. Family planning utilization in Pohnpei was examined for both adolescents and adult women of reproductive age using representative survey data collected in 2019. This study aimed to determine the prevalence of overall family planning use among adolescents and adults, prevalence of specific family planning methods, reasons for not utilizing family planning, and factors associated with family planning utilization.

## Methods

### Study Design

This study utilized a cross-sectional design. Survey data were collected in Pohnpei, Federated States of Micronesia in 2019.

### Population and Sample

There were two different study populations analyzed in this study: (1) adult women of reproductive age (18 to 49 years old) and (2) adolescents in high school (14 years and older). All Pohnpei residents 18 years and older were eligible for the Hybrid Survey, and the total sample size collected for the adult population was 1536, including 570 women of reproductive age (18–49 years old). Multi-stage sampling was used to recruit participants and consisted of geographical stratification for household sampling followed by the random selection of an individual from the household to participate. All high school students who were present on the day of the Rapid Youth High School Survey were eligible to participate, and 1726 students were surveyed.

### Study Variables

The main dependent variable of interest was family planning utilization. For adolescents, there was also an outcome of condom use during last intercourse. For adult women who used family planning to prevent pregnancy during the last sexual intercourse, a secondary outcome of interest was the type of family planning used. For adult women who did not use family planning during the last intercourse, we examined the reason for not doing so. For adolescents who did use a family planning during the last intercourse, a secondary outcome was type of family planning used. Independent variables for adolescents included demographic characteristics (age, gender, grade, and school type), behavioral factors (age first had sex, total number of sexual partners, tobacco use, betel nut use, and alcohol use), and BMI category. Independent variables for adult women similarly included demographic characteristics (age, education, marital status, religion, employment, and household income), indicators of healthcare access (annual visits, health insurance, cost as a barrier, ever had a clinical breast exam, and ever had a pap smear), behavioral factors (number of pregnancies, tobacco use, and alcohol use), and health factors (general health, depression or anxiety, diabetes mellitus, hypertension, and BMI category).

### Operational Definition of Variables

For both adult women and adolescents, utilization of family planning was defined as using any method to prevent pregnancy during the last sexual intercourse. Additional operational definitions can be accessed from the Hybrid Survey and Rapid Youth High School Survey (Pohnpei State Government Department of Health and Social Services, 2019; Cash, 2019).

### Study Instruments

For the first study population (adult women of reproductive age), data collected in the 2019 Hybrid Survey conducted in Pohnpei were utilized (Pohnpei State Government Department of Health and Social Services, 2019). The Hybrid Survey is a locally developed adult population-based survey that combines survey tools and includes questions specific to reproductive health that were asked of women aged 18–49 years (Cash et al., 2021). Data collection included a face-to-face interview, physical measurements of height, weight, and blood pressure, and biochemical measurement of fasting blood glucose and total cholesterol. For statistical analysis, women who were pregnant, trying to become pregnant, or who had a same sex partner were excluded (N = 121), leaving a sample of 449 women.

For the second study population (adolescents attending high school), data collected in the 2019 Rapid Youth High School Survey carried out in Pohnpei, Federated States of Micronesia were used (Cash, 2019). All high school students in Pohnpei who were present on the day of the survey were eligible to participate, with a total sample size collected of 1726 students. Data on substance use, nutrition, physical activity, and reproductive health were all self-reported by participants using a self-completed anonymous survey. Physical measurements of height and weight were used to calculate Body Mass Index (BMI). Reproductive health questions were asked of both male and female students on this survey.

### Data Analysis

SAS 9_4 64 bit was used for statistical analyses (SAS Institute, 2013). Basic descriptive statistics were conducted for all variables of interest. Chi-squared tests were conducted for all indicated independent variables by family planning utilization (usage of any method for adolescents and adult women; usage of condoms for adolescents). Variables were collapsed into fewer response categories than displayed in Table 1 to improve statistical power of chi-square tests. All findings with a p-value < 0.05 were considered statistically significant.

**Table 1:** Selected descriptive variables among the female reproductive age (18–49) adult population (N = 570)

Age category	N	%
18–24	120	21.1
25–34	157	27.5
35–44	190	33.3
45–49	103	18.1
Education		
Less than high school	245	43.0
High school completed	252	44.2
Associate’s degree completed	62	10.9
Bachelor’s degree completed	9	1.6
Graduate or professional degree completed	2	0.4
Marital status		
Single, never married	126	22.1
Married	414	72.6
Widowed	15	2.6
Divorced/separated	15	2.6
Religion		
Protestant	182	31.9
Catholic	322	56.5
Other	66	11.6
Employment		
Government employee	67	11.8
Non-government employee	83	14.6
Self-employed	109	19.1
Non-paid (volunteer, subsistence, etc.)	1	0.2
Student	46	8.1
Homemaker	104	18.3
Unemployed (able to work)	136	23.9
Unemployed (unable to work)	24	4.2
Household Income*		
< 5000	287	53.5
5000–10,000	169	31.5
> 10,000	81	15.1
Used family contraceptive during last intercourse (women not trying to get pregnant, N = 449)		
Yes	160	31.6
No	289	68.4

^*^33 individuals missing data on household income

### Research Ethics

Ethical issues were carefully considered and all data were deidentified to ensure anonymity of survey responses. All adult subjects who participated in the Hybrid Survey provided consent using a signed consent form. For the Rapid High School Survey, the Department of Education sent home a letter to all parents informing them of the high school survey and provided the opportunity for any parents to opt-out their child/children. High school adolescents then provided their verbal assent to participate in the survey. No IRB was completed for this research due to lack of IRB in FSM. Therefore, official approval for this research was received from the Pohnpei State Department of Health Services.

## Results

### Sample Characteristics

Characteristics of our first study population, reproductive age (18–49) adult women, can be found in Table 1 (N = 570). Most women either did not complete high school (43.0%), or their highest education completed was high school (44.2%). The majority of women were married (72.6%), and most were Protestant (31.9%) or Catholic (56.5%). Almost half of women (45.5%) reported being employed for wages. Over half of women (53.5%) had a household income of less than $5,000 per year. Overall, fewer than one out of three women of reproductive age (31.6%) who were not trying to get pregnant (N = 449) reported doing anything to prevent pregnancy during last intercourse.

Characteristics of our second study population, adolescents in high school, can be found in Table 2 (N = 1,726). The majority of students (57.7%) were female and about half (47.1%) were between 15–16 years old. Three out of four students (75.7%) attended public schools. About two out of five (38.0%) high school students reported ever having sexual intercourse. Overall, about one out of four (28.5%) high school students who were sexually active (N = 653) reported using any method to prevent pregnancy during last intercourse, and 22.6% reported using a condom at last intercourse.

**Table 2:** Selected descriptive variables among the adolescent high school population (N = 1726)

	N	%
Age*		
14	329	19.1
15 to 16	812	47.1
17 to 18	513	29.7
19 +	71	4.1
Gender		
Male	731	42.4
Female	995	57.7
Grade		
9	506	29.3
10	423	24.5
11	392	22.7
12	405	23.5
School type		
Public	1307	75.7
Private	419	24.3
Ever had sexual intercourse		
Yes	653	38.0
No	1067	62.0
Used any method to prevent pregnancy during last intercourse*		
Yes	185	28.5
No	465	71.5
Used condom during last intercourse*		
Yes	147	22.6
No	503	77.4

^*^1 student missing data on age, 3 students missing data on contraceptive use, 3 students missing data on condom use during last intercourse

### Bivariate Analysis

Table 3 examines the proportion of reproductive-aged adult women (excluding those who were trying to get pregnant, had a same sex partner, or were not sexually active) who used any method to prevent pregnancy during last intercourse by selected characteristics (N = 449). Young women (18–24 years old) had significantly lower prevalence of family planning use (21.7%) compared to women 25 to 39 years old (37.1%) and women over 40 (30.1%) (p = 0.021). Married women had a significantly higher prevalence of family planning use (36.0%) compared to unmarried women (18.6%) (p < 0.001). Women with health insurance also had a significantly higher prevalence of family planning use (38.6%) compared to women who did not have health insurance (28.7%) (p = 0.030). Women who have ever had a pap smear had a significantly higher prevalence of family planning use (43.4%) than those who have not ever had a pap smear (20.5%) (p < 0.001). Finally, as the number of previous pregnancies increased, so did family planning use prevalence: 14.5% of those with 0 pregnancies, 31.5% of those with 1 or 2 pregnancies, 35.8% of those with 3 to 5 pregnancies, and 41.0% of those with 6 or more pregnancies reported using family planning (p = 0.002).

**Table 3:** Selected characteristics stratified by contraceptive use among the female reproductive age (18–49) adult population* (N = 449)

	% Used contraceptive	p-value
Age category		
18–24	21.7	0.021
25–39	37.1	
40 +	30.1	
Education		
Less than high school	30.2	0.215
High school completed	30.1	
More than high school	40.9	
Marital status		
Married	36.0	< 0.001
Not married	18.6	
Religion		
Protestant	31.3	0.773
Catholic	30.9	
Other	35.6	
Employment		
Employed	31.4	0.935
Not employed	31.7	
Income		
< 5000	27.3	0.089
5000–10,000	34.9	
> 10,000	39.4	
General Health		
Excellent/very good/good	31.1	0.966
Okay/poor	31.3	
Ever had an annual visit		
Yes	33.2	0.170
No	26.6	
Health Insurance		
Yes	38.6	0.030
No	28.7	
Did not seek healthcare in the past 12 months because of cost		
Yes	30.9	0.783
No	32.1	
Depression or anxiety		
Yes	38.3	0.233
No	30.7	
Ever had a clinical breast exam		
Yes	42.9	0.070
No	30.2	
Ever had a pap smear		
Yes	43.4	< 0.001
No	20.5	
Number of pregnancies		
0	14.5	0.002
1 to 2	31.5	
3 to 5	35.8	
more than 6	41.0	
Used any tobacco in the past 30 days		
Yes	32.6	0.583
No	30.3	
Drank alcohol in the past 30 days		
Yes	37.7	0.210
No	25.0	
Diabetes mellitus		
Yes	30.8	0.871
No	31.7	
Hypertension		
Yes	30.4	0.838
No	31.7	
BMI category		
Underweight or normal weight	28.3	0.675
overweight	34.0	
obese	31.9	

^*^Women who were pregnant, trying to become pregnant, or who had a same sex partner were excluded (N = 121) from analysis

Table 4 examines the proportion of sexually active high school students who used any method to prevent pregnancy during last intercourse and condom use during last intercourse by selected characteristics (N = 653). There was a significant decreasing trend in condom use prevalence as age increased, where adolescents who were 14 years old were most likely to use condoms (36.1%) (p = 0.006). Males had a significantly higher prevalence of any family planning use (31.7%) and condom use (26.6%) than females (23.7% and 16.8%, respectively) (p = 0.026 and p = 0.004, respectively). Students who chewed betel nut had a lower prevalence of any family planning use (23.5%) than those who did not (35.1%) (p = 0.001). In addition, as BMI category increased, the prevalence of family planning use increased from 25.4% of those underweight or healthy weight to 37.8% among those who were obese (p = 0.032).

**Table 4:** Selected characteristics stratified by contraceptive use among the adolescent high school population who reported being sexually active (N = 653)

	% Used any method to prevent pregnancy	p-value	% Used condom	p-value
Age				
14	34.7	0.440	36.1	0.006
15 to 16	29.8		24.8	
17 to 18	26.5		17.4	
19 +	23.5		19.6	
Gender				
Male	31.7	0.026	26.6	0.004
Female	23.7		16.8	
Grade				
9	31.0	0.412	33.3	< 0.001
10	28.2		24.4	
11	32.2		25.3	
12	25.4		13.5	
School type				
Public	27.3	0.170	22.2	0.610
Private	33.6		24.4	
Age first had sex				
11 to 13	39.3	0.459	36.1	0.389
14 to 15	30.5		25.6	
16 or older	39.1		28.3	
Total number of sexual partners				
1 to 2	29.0	0.381	23.0	0.719
3 to 4	24.3		20.3	
5 or more	31.6		24.1	
Smoke tobacco				
Yes	26.6	0.344	22.0	0.751
No	30.0		23.1	
Chew betel nut				
Yes	23.5	0.001	20.0	0.061
No	35.1		26.2	
Any tobacco use				
Yes	26.5	0.219	22.0	0.667
No	30.9		23.4	
Drink alcohol				
Yes	28.0	0.742	21.9	0.606
No	29.2		23.6	
BMI category				
Underweight or healthy weight	25.4	0.032	20.9	0.294
Overweight	28.2		22.9	
Obese	37.8		27.7	

The frequencies of different methods used by reproductive-aged adult women during last intercourse (among those who reported using any method at last intercourse) are displayed in Table 5 (N = 156). The most commonly used type of family planning was shots (35.0%), followed by contraceptive implant (31.9%), and female sterilization (13.1%). The frequencies of different methods to prevent pregnancy during last intercourse used by adolescent high school students who are sexually active are shown in Table 6 (N = 186). The most commonly used type of family planning was condoms (68.3%), followed by withdrawal or other (14.5%), and birth control pills (12.9%).

**Table 5:** Type of contraceptive used among female reproductive age (18–49) adult population who reported contraceptive use during last intercourse (N = 156*)

Contraceptive type	N	%
Shots	56	35.0
Contraceptive implant	51	31.9
Female sterilization	21	13.1
Birth control pills	10	6.3
Other method	6	3.8
Male condoms	4	2.5
Rhythm method	3	1.9
Withdrawal	2	1.3
Contraceptive ring	2	1.3
Male sterilization	1	0.6
Intrauterine device (IUD)	0	0.0
Contraceptive patch	0	0.0
Diaphragm	0	0.0
Female condom	0	0.0
Emergency contraception	0	0.0

^*^4 individuals missing data on contraceptive type

**Table 6:** Type of contraceptive used among the adolescent high school population who reported using contraceptive during last intercourse (N = 186)

Contraceptive type	N	%
Condoms	127	68.3
Withdrawal or other	27	14.5
Birth control pills	24	12.9
Shot, patch, or birth control ring	8	4.3
IUD or implant	0	0.0

Among women who did not use a method to prevent pregnancy during last intercourse (N = 340), frequencies of reasons for not using family planning during last intercourse are displayed in Table 7. The top reason for not using family planning was “You just didn’t think about it” (26.5%). Other popular responses included female sterilization (16.8%), not thinking they were going to have sex (10.3%), they want to become pregnant (9.3%), and they don’t care if they get pregnant (7.9%).

**Table 7:** Reason for not using contraception among female reproductive age (18–49) adult population who reported not using contraceptive during last intercourse (N = 340)

Reason	N	%
You just didn’t think about it	90	26.5
You had tubes tied (sterilization)	57	16.8
Other reason	50	14.7
You didn’t think that you were going to have sex	35	10.3
You want a pregnancy	31	9.1
Don’t care if you get pregnant	27	7.9
You or your partner don’t want to use birth control	15	4.4
You or your partner don’t like birth control/side effects	14	4.1
Lapse in use of method	8	2.4
Don’t think that you could get pregnant	5	1.5
You had a hysterectomy	2	0.6
You had a problem getting birth control when you needed it	2	0.6
Religious reasons	2	0.6
You are currently breastfeeding	1	0.3
You couldn’t pay for birth control	1	0.3
Your partner had a vasectomy	0	0.0
You just had a baby/postpartum	0	0.0
You are pregnant now	0	0.0
Same sex partner	0	0.0

## Conclusions

This study is the first to characterize family planning utilization and related variables in FSM. In addition to describing family planning utilization, we give several recommendations on how to increase access to and utilization of family planning resources.

In the United States, 64.9% of women of reproductive age (15–49 years old) currently use family planning (CDC, 2018). In contrast, 31.6% of women 18–49 years old in Pohnpei reported using family planning. Women who are young and/or unmarried reported utilizing family planning significantly less than older and/or married women. This is consistent with previous findings that young, unmarried women in low- or middle-income countries tend to have less access to family planning (Chandra-Mouli et al., 2014). In addition, we found that family planning utilization is significantly associated with factors that are indicators of healthcare access, including health insurance and whether a woman has ever had a pap smear. Although the results were not significant, women with a higher family income and those who have had an annual exam in the past year reported the use of family planning at a higher prevalence. Similarly, family planning use stratified by ever having a clinical breast exam is insignificant, but the trend indicates that women who have had a breast exam were more likely to use family planning (42.9%) than those who had not (30.2%). Women who had more previous pregnancies had a significantly higher prevalence of family planning utilization, likely due to the desire to limit their family size.

Similarly, 71.5% of high school age, sexually active adolescents in Pohnpei reported not using any family planning during last sexual intercourse. In 2019 in the United States, only 11.9% of sexually active adolescents reported not using any family planning during last sexual intercourse (Centers for Disease Control & Prevention, 2019). We found that the prevalence of condom usage for boys was significantly higher than the prevalence of condom usage for girls, and condom usage decreased as age increased. Condoms are readily available at most high schools in Pohnpei, and there is less stigma for boys around using condoms than for girls. In agreement with previous research, family planning use in youth may be associated with risky behaviors (Pinhey & Wells, 2007). Adolescents who chewed betel nut were significantly less likely to use any family planning during last sexual intercourse, and although not significant, adolescents who smoked tobacco, used any tobacco, and drank alcohol reported using family planning at lower rates than those who did not. However, there is no clear relationship between family planning use and the age of first sexual intercourse or the total number of sexual partners.

Based on the finding that young, unmarried women are using family planning at low rates, interventions to increase utilization of family planning to promote healthy families should target this group. The most common reason that women reported for not using family planning was that they simply didn’t think about it, so it may be important to ensure access to family planning methods that do not require consistent thought, such as shots, contraceptive implants, or intrauterine devices (IUDs). These methods were found to be underutilized in the high school adolescent population. Currently, there is no required sex education curriculum at schools in Pohnpei State. Previous research has found that adolescents who receive sex education are significantly more likely to use family planning, and sex education is also associated with delaying the age of sexual initiation and first pregnancy (Lindberg & Maddow-Zimet, 2012). By providing students with education on family planning, adolescents would be able to make informed decisions on when to have sex and whether or not to use family planning.

Another factor that should be considered when planning an appropriate intervention is stigma and the general attitude toward family planning utilization in Pohnpei. Currently, the misconceptions in the Pacific Islands region around family planning, especially for adolescents, serve as a barrier to increasing the uptake of family planning (White et al., 2018; Winn-Dix et al., 2016). In addition to improving access to family planning resources, it is important address these cultural barriers and familial factors to improve utilization (Bongaarts & Bruce, 1995). Previous research has found that men’s attitudes toward family planning are a better indicator of family planning utilization than women’s attitudes in Micronesia (Brewis, 2001). Future interventions should target both men and women at a young age. While many Pohnpeian women do not speak with their healthcare providers about family planning until after they have had their first child, it may be important to initiate these conversations earlier—during routine well woman visits or cancer screenings, for example. Therefore, there may be an opportunity to join efforts on increasing prevalence of annual well woman visits with increasing education on family planning. While increased education of and access to family planning does not mean someone must use family planning, having the option provides many potential benefits—decrease in maternal deaths, improved health outcomes for children, and improved educational and economic outcomes for women, although this relationship has not been specifically studied in FSM (Ahmed et al., 2012; Cleland et al., 2012; Darroch et al., 2016). It can also minimize unintended pregnancy and the spread of sexually transmitted infections (WHO, 2022).

This novel study characterized family planning utilization in adult reproductive-aged women and adolescents in Pohnpei. A strength of this study was the survey design—both the Rapid Youth High School Survey and the Hybrid Survey were administrated by trained individuals and had high response rates. The Rapid High School Survey included all high schools in Pohnpei, so is highly representative of Pohnpei high school students. The Hybrid Survey collected data from a large representative sample of overall adults residing in Pohnpei. One weakness is the small sample sizes, which is simply due to the small population of Pohnpei. Another is that the Rapid Youth High School Survey includes adolescents over the age of 18 who would also be eligible for the Hybrid Survey. This could have led to some individuals surveyed twice and being present in both samples. However, this number is small enough that it should not greatly affect our results. Additionally, the Rapid High School Survey does not capture out of school youth, who may be at higher risk for some behaviors being examined in this study. Finally, some responses were self-reported, which may have introduced response bias, especially for questions on sensitive topics such as sexual behavior. One major limitation is the descriptive nature of this study that does not allow us to control for confounding factors. Additional analytical studies are needed to further understand how multiple factors together are associated with family planning in Pohnpei.

In Pohnpei, the prevalence of family planning utilization is low for both adolescents and adult reproductive-aged women, and it is especially low for girls and women who are young and/or unmarried. These findings indicate the need for a better understanding of obstacles to family planning utilization and possible interventions that target both access and cultural barriers.

## Data Availability

Data used is available through published reports.

## Code Availability

SAS code was used and can be shared upon request.
